# Use of Hydrogels in Regenerative Medicine: Focus on Mechanical Properties

**DOI:** 10.3390/ijms252111426

**Published:** 2024-10-24

**Authors:** Flavia Carton, Manuela Rizzi, Elena Canciani, Gianluca Sieve, Dalila Di Francesco, Simona Casarella, Luca Di Nunno, Francesca Boccafoschi

**Affiliations:** 1 Department of Health Sciences, Università del Piemonte Orientale, 28100 Novara, Italyelena.canciani@uniupo.it (E.C.); simona.casarella@uniupo.it (S.C.);; 2 Laboratory for Biomaterials and Bioengineering, CRC-I, Department of Min-Met-Materials Engineering, University Hospital Research Center, Regenerative Medicine, Laval University, Quebec City, QC G1V 0A6, Canada

**Keywords:** hydrogel, tissue engineering, biocompatibility, regenerative medicine, biomaterials, tissue mechanical properties

## Abstract

Bioengineered materials represent an innovative option to support the regenerative processes of damaged tissues, with the final objective of creating a functional environment closely mimicking the native tissue. Among the different available biomaterials, hydrogels represent the solution of choice for tissue regeneration, thanks to the easy synthesis process and the highly tunable physical and mechanical properties. Moreover, hydrogels are biocompatible and biodegradable, able to integrate in biological environments and to support cellular interactions in order to restore damaged tissues’ functionality. This review offers an overview of the current knowledge concerning hydrogel synthesis and characterization and of the recent achievements in their experimental use in supporting skin, bone, cartilage, and muscle regeneration. The currently available in vitro and in vivo results are of great interest, highlighting the need for carefully designed and controlled preclinical studies and clinical trials to support the transition of these innovative biomaterials from the bench to the bedside.

## 1. Introduction

Due to population aging, life expectancy is increasing, together with a physiological condition associated with the need for therapeutic solutions aimed at preserving or restoring tissues and organ functionalities following age-related degeneration. To date, regenerative medicine and tissue engineering represent an innovative approach to this still unmet clinical need, as well as to the lack of donors for organ transplantation [1]. In particular, tissue engineering, by using a combination of biomaterials, cells, and bioactive molecules, allows for the development of functional biological structures closely mimicking specific organs and tissues that could be used to restore the damaged structure’s biological integrity.

Hydrogels represent one of the most commonly used biomaterials for tissue engineering. They are three-dimensional networks composed of hydrophilic polymers able to absorb and retain significant amounts of water or biological fluids while maintaining their spatial structure. The most relevant features that make these materials excellent candidates for hierarchically organizing cells into tissue-like structures are their extraordinary biocompatibility, biodegradability, and rheological and mechanical properties. Indeed, to ensure their physiological activity and avoid the possible side effects resulting from the stress shielding mechanisms, it is desirable to develop materials with mechanical properties comparable to the native tissues [2].

This review summarizes tissue biomechanical characteristics in relation to hydrogels used in regenerative medicine, with special attention to skin, bone, cartilage, and muscles.

## 2. Mechanical Properties of Human Tissues and Organs

The mechanical properties of biological tissues are strictly dependent on the anatomical localization, as well as on the extracellular matrix (ECM) composition and microstructure, which are essential in driving cellular behavior, by providing specific extracellular signals; thus, mimicking the mechanical properties still represents a great challenge in the development of successful tissue-engineered substitutes [3,4].

In this context, it is of great importance to consider that, in each different tissue, cells are also guided by different mechanical stimuli (mainly represented by shear, compression, and tensile stress) of both external and local origin. These different stressors determine a deformation response that varies according to the considered tissue’s stiffness [5]. The tissue’s stiffness is commonly expressed in terms of elastic or Young’s modulus and varies according to the tissue’s functionality. In fact, it is the key parameter involved in defining tissue structure, as well as its ability to provide structural support to the tissues as well as to organs. Table 1 briefly summarizes the degree of stiffness of different tissues [6].

The following sections offer a brief overview of the biomechanical features of skin, bone, cartilage, and muscles, with the aim of highlighting the key parameters to be considered when developing biological substitutes.

### 2.1. Skin

The skin is composed of three layers: epidermis (external layer), dermis (middle layer), and hypodermis (deep layer), and, being the largest organ of the human body, represents the first barrier against environmental pathogens. This organ normally deals with constant mechanical tension, including deformation and compression caused by the body’s movement and by the forces arising from the underlying dermal layers; the skin’s ECM is composed of a crosslinked network of laminins, elastic fibers, collagens, and proteoglycans (PGs), such as perlecan and nidogen [7,8,9]. Skin ECM components, altogether, contribute to dynamically modulating the swelling ability as well as the structural stiffness. In this context, it is important to consider that collagens, which are mainly represented by type I (accounting for 60–80% of the total amount) and type III (accounting for 15–20% of the total amount), assure tissue stiffness, while the elastic components provide skin physiological elasticity, and glycosaminoglycans (GAGs) account for its hydration, thanks to their ability to entrap large amounts of water [10,11,12,13,14,15,16,17,18].

The typical dynamic adaptability of the skin to different mechanical stimulations relies on the collagenous and elastic components of the ECM. It has been observed that, when a strain stimulation of up to 0.3% is applied, elastin fibers offer low resistance, while collagen fibers remain entangled and intertwined, not contributing to the stiffness of the tissue. On the contrary, when mechanical stress increases, it is dissipated by collagen fibers that linearize and stretch, resulting in a localized increase in tissue stiffness. A further increase in strain stimulation, reaching values in the 0.6–0.7% range, results in a linear stress–strain response. Finally, when the applied strain–stress overcomes the value of 0.7%, collagen fibers start to degenerate, resulting in a progressive loss of the skin’s physiological mechanical properties [19]. As a matter of fact, detailed knowledge of skin mechanical behavior is essential to discriminate between healthy and pathological tissues and to design effective biomaterials for regenerative purposes.

### 2.2. Bone Tissue

Bone tissue is a stiff connective tissue representing the basic element of the skeletal system, where it has to withstand both compressive and tensile stresses as well as bending and torsion stimuli. The ability of this tissue to support such mechanical stresses is closely related to its composition [20]. Bone mineral constituents effectively counteract compressive forces, thus assuring tissue hardness, while collagen fibers, mainly represented by type I (accounting for 90–95% of the total amount) and types III and V (present in small amounts), assure its flexibility and elasticity. Moreover, PGs and GAGs side chains play an important role in bone biomechanics by regulating the hydrostatic and osmotic pressure as well as the poroelastic behavior of the mineralized tissue [17,21,22,23].

The mechanical properties of bone tissue are not only dependent on ECM composition, as it is well known that its structural features (e.g., fiber orientation, trabecular architecture, mineralization, and porosity) also play a key role in determining these properties. From a structural point of view, bones are composed of stacked tissues, namely the cortical bone (representing the outer layer, with a porosity ranging from 5% to 15%) and the trabecular bone (representing the inner part, with a porosity ranging from 40% to 95%) [24,25]. The cortical bone represents the most compact structure, showing a higher degree of toughness, stiffness, and strength; in response to a compressive force, it slightly deforms, quickly breaking down following increased stimulation [26,27]. Such mechanical behavior is also affected by cortical porosity, as this parameter negatively correlates with the tissue elasticity, by its compressive ultimate stress response, and by fracture toughness, finally accounting for the tissue-specific elastic modulus and yield stress [24,28,29,30,31]. On the other hand, the trabecular bone is a spongy structure that undergoes active remodeling and shows a more ductile behavior towards compression forces, thus leading to larger deformations following small compressive stimuli compared to cortical bone [32,33].

Considering that bone structure and properties could be affected both by pathological processes and physiological aging, the understanding of their specific mechanical features is essential for properly restoring bone integrity.

### 2.3. Cartilage

Articular cartilage (AC) is a load-bearing tissue able to mitigate compressive forces in the joint and limit friction that could damage the underlying bone. These abilities are due to its highly hydrated ECM, composed of PGs, collagens (mainly represented by type II (accounting for 90–95% of the total amount), type IX (accounting for 1–5% of the total amount) and XI (accounting for 1–5% of the total amount)), and non-collagenous proteins and glycoproteins [17,34,35,36,37]. The AC’s ECM microstructure is once again the major responsible for the whole tissue’s mechanical properties. In particular, the superficial layer (10–20% of the total AC) is a highly hydrated structure composed of tightly packed collagen fibers with parallel orientation, thus allowing tissue deformation when subjected to external stress. The middle layer (40–60% of the total tissue), instead, is rich in PGs, and it is characterized by thicker collagen fibers randomly oriented, thus representing the first line of resistance against compressive forces. The deep layer (approximately 30% of the total AC) is composed of PGs and thicker collagen fibers with a perpendicular orientation, thus providing the greatest resistance to compressive forces. Finally, the calcified layer anchors the deep layer of collagen to the underlying bone, thus offering excellent resistance to shear forces [34,35,36,38,39,40].

Concerning the mechanical properties, the AC is a viscoelastic material able to sustain transient deformation: when a compressive force is applied to the cartilage, the pressure of the interstitial fluid increases, causing the fluid to flow out of the ECM, which then returns into the interstitial space when the compressive force is removed. This fluid behavior is the main reason for the force dissipation, while only a small part is dissipated through ECM deformation. Moreover, when compression occurs, the electrostatic repulsion between negatively charged PGs increases, resulting in an increased tissue stiffness against compression [36,41,42,43,44]. Experimental studies focusing on cartilage mechanical properties highlighted the ability of this tissue to tolerate a compressive strain of up to 30% due to its compressive stiffness resulting from a non-linear stress–strain behavior [45,46]. Moreover, considering the natural inhomogeneity of the tissue, it should be noted that mechanical properties depend on the considered tissue layer; the majority of the available studies are focused on the external one without considering the existing variations between the different layers [3]. Interestingly, recent research focused on the characterization of the mechanical responses of the different cartilage layers [47,48], highlighting the presence of a gradient in the tissue-specific elastic modulus between layers [3]. Considering that AC mechanical properties are affected by age-related pathological degeneration, a deep knowledge of the tissue’s microstructure is essential for developing bioengineered matrices for cartilage regeneration.

### 2.4. Muscles

The muscular tissue’s distinctive characteristic is represented by contractility. In vertebrates, three types of muscular tissues can be identified, indicated as skeletal, cardiac, and smooth muscles, each one showing specific microstructural features that directly affect their own mechanical properties.

The skeletal muscle is a viscoelastic tissue composed of long myofibers associated with tendons, which undergo neurogenic contraction [49]. The skeletal muscle’s mechanical behavior strongly depends on the associated ECM, which is organized in different layers: endomysium, surrounding each myofiber; perimysium, grouping muscle fibers in fascicles; and epimysium, wrapping the whole muscle. Endomysium and perimysium are the layers mostly involved in defining the skeletal muscle’s mechanical features [50]. Endomysium is composed of randomly arranged collagen fibers, while perimysium is composed of hierarchically organized collagen fibrils. As observed for bone and cartilage, skeletal muscle connective tissue is also composed of different types of collagens, differently represented in terms of percentage: collagen type I and type III fibers account each for roughly 3% of the total collagen of the tissue, while the others (mainly represented by types IV and V) account for about 2% of the total. Overall, these play an important role in sustaining the tissue’s biomechanical behavior, undergoing conformational changes during muscle elongation, and contributing to the transmission of muscle contractile force to the bones through the tendons [50,51,52,53,54,55,56].

The cardiac muscle is an elastic tissue composed of specific cells able to generate myogenic contractions, whose stress–strain behavior is mainly dependent on its ECM composition. In particular, it has been observed that, during diastole, pericardial collagen fibers are uncoiled, thus offering low resistance to the stretch and allowing ventricular filling, while during systole they linearize, transmitting force and helping to maintain myocyte alignment throughout the cardiac wall [57]. Also, in cardiac ECM, different components collaborate in defining the tissue’s mechanical properties: collagens are mainly represented by type I (accounting for 85% of the total amount), type III (accounting for 15% of the total amount), and type V (accounting for up to 5% of the total amount) fibers are mainly involved in contraction; elastin is known to support tissue-specific elastic properties by interweaving with collagen fibers; and PGs and GAGs are involved in fluid movement regulation during systole [58,59,60,61,62,63].

The smooth muscle is a non-striated muscular tissue characterized by myogenic contraction, mainly localized in the visceral organs, where its stress relaxation responses are essential in ensuring body functions. Also, in this context, the specific tissue’s mechanical features strongly depend on ECM organization, as the application of tensile and compressive forces, as well as shear stress, result in matrix remodeling and finally, in the maintenance of tissue homeostasis [64,65].

## 3. Hydrogels in Tissue Engineering and Regenerative Medicine

In regenerative medicine applications, matrices are essential to sustain tissue integrity restoration by providing a 3D structure for cellular functions. Thanks to their highly hydrophilic 3D structure, which is essential in allowing the diffusion of biological fluids, cells, and metabolites, hydrogels are one of the most used biomaterials in the field of regenerative medicine [1,66]. These materials exhibit viscoelastic characteristics that can be easily tuned by adjusting processing conditions (e.g., crosslinking rate, gelation conditions, temperature, pH, etc.), in order to closely mimic the native tissue characteristics, thus providing structural and biochemical support to the surrounding cells [67]. Moreover, it is noteworthy that hydrogels, once implanted into the appropriate body district, should integrate with the existing tissues, finally undergoing natural degradation when tissue regeneration is reached.

### 3.1. Hydrogels: Characteristics, Classification, and Production

Hydrogels can be obtained from synthetic or natural polymers, or as a hybrid of both. Table 2 shows the most common hydrogels used to support tissue regeneration, highlighting the known pros and cons.

Regardless of their nature, hydrogels can also be tailored to allow their adaptability to the surrounding environment. Such innovative scaffolds, called “smart hydrogels”, not only undergo the classical swelling processes but can also modify their behavior according to specific external stimuli like temperature, pH, or electric field. Among them, the most studied in the biomedical field are the temperature and/or pH-responsive materials, as they allow a more efficient integration in the surrounding environment [1,68].

Because one of the major advantages of hydrogel scaffolds is represented by their highly tunable characteristics, special attention should be paid to their microstructure, which mainly depends on substrate crosslinking. Based on the crosslinking procedure used during their synthesis, hydrogels could be classified as physical or chemical hydrogels [1,68]. In particular, in physical hydrogels, the polymer chains’ crosslinking is based on non-covalent forces, resulting in weak and reversible interactions, which can lead to, under specific conditions, matrix dissolution in water or organic solvents. The use of non-toxic crosslinking molecules and their limited lifetime in biological environments make these scaffolds a safe choice for clinical applications. On the other hand, chemical hydrogels are characterized by strong covalent bonds, which result in stable networks even in the swollen state. Moreover, the use of the chemical crosslinking approach allows a fine-tuning of the final matrix mechanical properties and biodegradability [1,68].

The choice of crosslinking is made according to the hydrogel’s desired properties, which are dependent on the final application. Considering that the crosslinking approach strongly depends on the final application of the scaffold, the main parameters that have to be considered for preparing hydrogels are (I) the amount of water the matrix is expected to absorb, and (II) the binding of the polymer chains within the gel network. The swelling parameter, which can be regulated through the starting polymer molecular weight and/or charge, as well as through the crosslinking density, represents a critical factor because it influences the solute diffusion coefficient, the surface wettability and mobility, and the optical and mechanical properties of the scaffold.

#### 3.1.1. Hydrogels Fabrication Techniques

In order to produce hydrogel-based scaffolds suitable for biomedical applications, several fabrication techniques have been developed: (I) emulsification, (II) freeze-drying, (III) porogen leaching, (IV) gas foaming, (V) electrospinning, (VI) three-dimensional printing, (VII) photolithography, and (VIII) sol–gel technique [1,73,74,75,76,77,78,79,80]. Table 3 briefly summarizes the key features of these fabrication methods.

#### 3.1.2. Mechanical Properties of Hydrogels

To achieve the generation of “artificial tissues” with mechanical performance comparable to those of native tissues, it is essential to deeply understand hydrogel mechanical properties. Theoretical approaches allow the prediction of the final polymer structure as well as the estimation of the expected elasticity, taking into account the nature of the substrate, the crosslinking procedure, and the biological environment of the implant [81]. Considering their inhomogeneous structure, in the swollen state hydrogels show poor deformability, as demonstrated by the experimental evaluation of their responses to shear or compression stresses [82]. Another critical parameter that is usually evaluated through an experimental approach is the elastic modulus, defined as the relationship between the applied stress (force per area unit) and the axial strain (proportional deformation). In native tissues, the elastic modulus strictly depends on their function and localization, with higher values corresponding to greater stiffness [83].

Technically, standard methods to evaluate hydrogel viscoelastic properties work under shear strain, where different loads are applied to the material under a constant extension rate. Such measurements clearly show that hydrogel mechanical properties can be finely regulated by modifying the nature and concentration of the starting substrates, the crosslinking approach and density, as well as the scaffold–composition ratio [1].

Furthermore, it is worth noting that scaffold mechanical properties deeply influence cell adhesion, migration, proliferation, and even differentiation [1,83,84], as observed with human mesenchymal stem cells (hMSC), which undergo neurogenic differentiation when cultured on hydrogels with low stiffness and myogenic or osteogenic differentiation when cultured on scaffolds with higher degrees of stiffness, close to the ones observed in the native tissues [85].

#### 3.1.3. Hydrogel/Tissue Mechanotransduction

Tissue regeneration is a dynamic process in which biomechanical signaling plays a key role in defining tissue homeostasis and repairing processes, as it is known that shear stress, tension, and compression forces provide crucial information to modulate cellular physiological behavior and homeostasis. In this context, hydrogels provide a template, mimicking the native extracellular matrix and providing the cells with biological and mechanical stimuli needed to support the regeneration process. In particular, biomechanical signals can originate both from inside and outside the cells and finally result in the control of biological and cellular responses through the conversion of a pure mechanical stimulus into a biochemical signal [6,86,87].

Mechanotransduction allows cell adaptation to a dynamic mechanical environment and relies on molecular conformation changes in key cellular components, such as cell-cell junctions (e.g., adherens and tight junctions, desmosomes), cell-matrix junctions (e.g., hemidesmomes, focal adhesions), mechanosensitive ion channels and G protein-coupled receptors, as well as transcription factors (e.g., YAP/TAZ), and intracellular signaling pathways (e.g., Wnt/β-catenin). Table 4 summarizes the most important cellular actors involved, sensing mechanical stimuli both at the cell membrane level and intracellularly [86,87,88,89,90].

## 4. Hydrogels in Regenerative Medicine

Different hydrogels have been developed to support the regeneration of damaged tissues or organs. In particular, these novel materials have been proven to mimic the native ECM biomechanics and to support cell survival, spread, and differentiation while assuring metabolic exchanges. Another essential feature that should be achieved to sustain their use as regenerative support is represented by their biodegradability, allowing their replacement by the newly formed matrix at the end of the regeneration process. To date, several hydrogels have been developed and characterized to support the regeneration of different human tissues, such as skin, bone, cartilage, and muscle.

### 4.1. Hydrogels for Wound Care

Skin damages can be classified into acute wounds (mechanical injuries, chemical injuries, surgical wounds, etc.) and chronic wounds (burns, infections, diabetes, etc.) that can be treated with several medical devices such as gauzes, films, foams, nanofibers, hydrocolloids, and hydrogels [91,92,93]. Among the currently available solutions, hydrogels have gained attention thanks to their ability to enhance wound regeneration while providing a moist environment. Moreover, hydrogels can be loaded with anti-microbial agents, anti-inflammatory drugs, bioactive molecules, and nanoparticles, thus enhancing the wound healing processes [94,95]. In fact, a major challenge in wound healing is represented by fighting bacterial infections, which can significantly impair the healing process. A common strategy to face this problem is represented by the use of active antimicrobial agents such as antimicrobial peptides, antibiotic drugs, or antibacterial materials in the currently available wound dressings. Several researchers have successfully tested, both in vitro and in vivo, hydrogels incorporating antimicrobial molecules, such as amphotericin B (Amb) and antioxidant materials like curcumin and t-resveratrol [96,97], as well as thermo-responsive drug carriers containing antibiotics [98], antibacterial nanoparticles [94] or antimicrobial peptides (AMPs) [93,94,99,100]. Finally, another successful approach is represented by the use of materials having intrinsic antibacterial activity, such as chitosan, to obtain hydrogels [101].

Another critical step in wound healing is represented by the sustained inflammatory response of the damaged district. Hydrogels represent an interesting approach to dealing with this issue, as it is known that their intrinsic physical and chemical properties can modulate tissue immune response [97].

In this context, while the easier approach is represented by the use of hydrogels derived from natural compounds, many research groups developed differently functionalized materials to improve their immunomodulatory potential. In fact, immunomodulating hydrogels have been successfully obtained by adding specific functional groups (i.e., peptides, growth factors, cytokines, and antibodies), but also by using the biomaterial to deliver mesenchymal or adult stem cells to the site of injury to support the healing process [93,101,102,103,104,105].

In addition to the above-discussed devices showing antibacterial as well as immunomodulatory properties, recently, mechanically responsive hydrogels have been successfully developed [106,107,108]. To physiologically support the wound healing process, such novel matrices should be able to modify their shape, thus allowing wound contraction [109,110]. Finally, the greatest challenge in developing scaffolds able to mimic skin mechanical properties is to produce a complex matrix reproducing the stiffness of the different cutaneous layers, whose feasibility has been proven by the pioneering work of Ma and coworkers, who developed a 3D bioprintable gelatin–alginate hydrogel characterized by a stiffness gradient able to guide stem cell infiltration and tissue remodeling [111]. These works highlight the importance of evaluating and tuning the mechanical component in hydrogel scaffolds for skin wound healing, as they also greatly contribute to the success of tissue regeneration.

**Figure 1:**
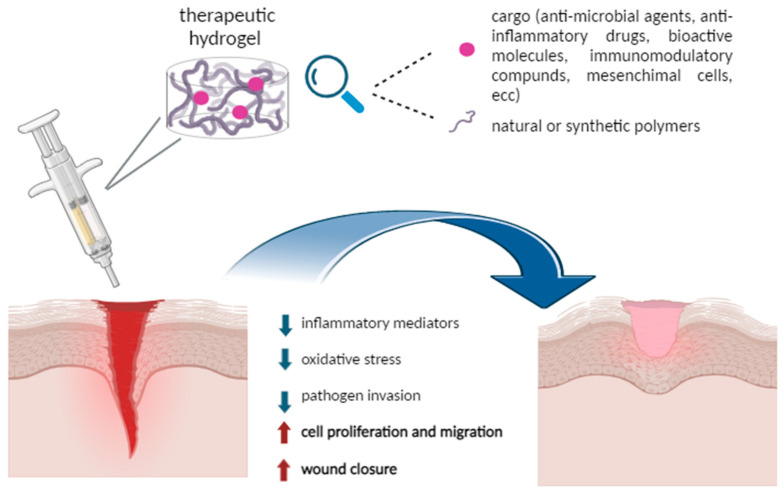
Schematic representation of hydrogel performance in supporting wound healing. Image created with BioRender.com.

Figure 1 summarizes the main features involved in the hydrogel-sustained wound healing process.

To date, in the field of skin regeneration in response to wounds, many goals have been achieved, as demonstrated by the successful production of hydrogels with antibacterial activity, immunomodulatory potential, and the ability to support wound contraction thanks to their intrinsic mechanical properties. Nevertheless, other important challenges need to be addressed in order to obtain superior solutions for wound management. Among them is the need to develop scaffolds able to control the oxygen supply at the wound site, as it is known that oxygen is also a crucial factor in wound healing. Another important feature in wound healing that leads to a new challenge in wound dressing development is scar formation and the lack of natural skin appendages (e.g., hair follicles, sebaceous, and sweat glands) regeneration. Several promising results come from the work of several researchers, who developed innovative hydrogels able to provide a scarless environment able to support skin appendage regeneration. Lastly, it is also important to remember that, in some cases, the wound-healing process could be associated with a dysregulation of cellular and biological responses, leading to non-healing or difficult-to-heal wounds, supporting the need for continuous innovation in the field of growth factor-loaded scaffolds in order to obtain solutions able to coordinate cellular responses in tissue repair. All the above-mentioned challenges are nowadays addressed by many research groups with promising results, thus fostering the development of skin substitutes able to restore not only skin functionality but also aesthetics (e.g., pigmentation, appendages), leading to a customized solution for personalized treatment [112,113].

### 4.2. Hydrogel for Bone Regeneration

Bone can be damaged following mechanical insults (i.e., trauma or fractures) or pathological degeneration (i.e., arthritis, infections, cancer, osteoporosis, or inflammation). In order to restore tissue integrity, the most common approaches are represented by allografts and autografts. Despite representing the current gold standard in clinical practice, these approaches show several drawbacks, such as limited availability, graft failure, and immune rejection. To overcome bone grafting limitations, the development of bioengineered materials able to support tissue regeneration represents a promising solution [114].

Bone healing is a complex physiological process characterized by three consecutive phases: inflammation, osteogenic differentiation, and bone remodeling. Bioengineered scaffolds should provide a microenvironment able to support the osteogenic process. To this extent, attention should be paid to vascularization, as it is well known that osteogenesis and angiogenesis are two biological processes that are strictly coupled, with VEGF (vascular endothelial growth factor) playing a key coupling role, finally assuring faster vascularization and improved bone deposition, as demonstrated, in a rat model, by the superior regenerative ability of an osteogenic differentiated mesenchymal and endothelial cells bearing scaffold compared to the scaffold alone [23,115,116,117]. Furthermore, it should be considered that an ideal scaffold needs to closely mimic the mechanical and physical properties of the outer as well as the inner layers of the bone [118]. To achieve such a crucial result, it is essential that ions like calcium (Ca^2+^), magnesium (Mg^2+^), and zinc (Zn^2+^) could be directly integrated into biomaterials to enhance their regenerative properties while improving their mechanical characteristics [119]. Ion-doped hydrogels for bone regeneration have been successfully developed by different research groups, such as Zhang and colleagues [120] who developed a novel magnesium ion-incorporating dual-crosslinked hydrogel able to promote osteogenic differentiation and angiogenesis in both in vitro and in vivo models. Chen and coworkers also developed a protein-crosslinked hydrogel able to deliver and guarantee optimal Mg^2+^ and Zn^2+^ concentrations, resulting in a synergistic enhancement of bone restoration by modulating the MAPK signaling pathway [121].

An alternative approach to hydrogel doping with mineral ions is represented by the incorporation of bioceramic nanocomposites into the scaffold. One of the most used bioceramic materials in this context is hydroxyapatite, which has been proven to increase local Ca^2+^ concentrations, resulting in MSC growth and differentiation and osteoblast proliferation [122]. The feasibility of this approach has been demonstrated by the successful development of a biodegradable poly(2-hydroxyethyl methacrylate) (PHEMA)-gelatin hydrogel containing nanohydroxyapatite dropped with metal ions showing considerable elasticity and high mechanical strength [123].

Promising results were also obtained by Liang and colleagues, who obtained an osteogenic microenvironment by combining biomimetic hydrogels of periosteum-decellularized extracellular matrix and calcium phosphate oligomers crosslinked with nano-hydroxyapatite (nano-HAP) [124]. Furthermore, Vitale’s research group developed a nanocomposite fluorenylmethoxycarbonyl-based RGD-functionalized peptide hydrogel modified with hydroxyapatite nanopowder (Hap) showing improved rheological properties and the ability to sustain osteoclasts (OCs) differentiation and function [125]. Finally, hydroxyapatite ball-flower particles (OHAHs) have been successfully encapsulated in hydrogels intended for bone repair [126].

Hydrogels that could be successfully used to support bone regeneration not only have to assure a pro-osteogenic microenvironment but also need to display mechanics comparable to the native tissue, as has been demonstrated by the increased proliferation and differentiation rates observed in cells grown on matrices closely mimicking bone tissue [127]. To achieve this goal, a promising strategy is represented by the use of double network hydrogels, novel structures characterized by high fracture strength and wear resistance associated with a low coefficient of fraction that can be obtained through a dual crosslinking approach [128,129].

In the context of bone regeneration, hydrogels have also been investigated as cell delivery systems. As a matter of fact, bone-specific cells such as pre-osteoblasts and osteoclasts have been successfully encapsulated within the hydrogel matrix [125,130], as well as MSCs that, thanks to their extraordinary differentiation potential, could represent a very promising solution to support new tissue formation [131,132].

To guarantee proper bone regeneration, another crucial step is represented by immunomodulation, allowing immune cells to switch from a pro-inflammatory to a pro-regenerative phenotype. To achieve this goal, Wang and colleagues developed an oxidized glucomannan (GM) hydrogel grafted with RADA16 peptide able to induce macrophage polarization toward the M2 phenotype, thus reducing the inflammatory response while sustaining the healing process [133]. Hydrogels with immunomodulatory properties have also been developed by other researchers, paving the way for the use of bioengineered hydrogels in the treatment of bone defects of different clinical natures [134,135,136].

Finally, it should be considered that hydrogels could also represent an effective drug delivery system that has already been successfully tested in osteosarcoma and chronic osteomyelitis targeted therapy, thus expanding the range of the possible clinical applications of the newly developed bioengineered hydrogels [137,138].

**Figure 2:**
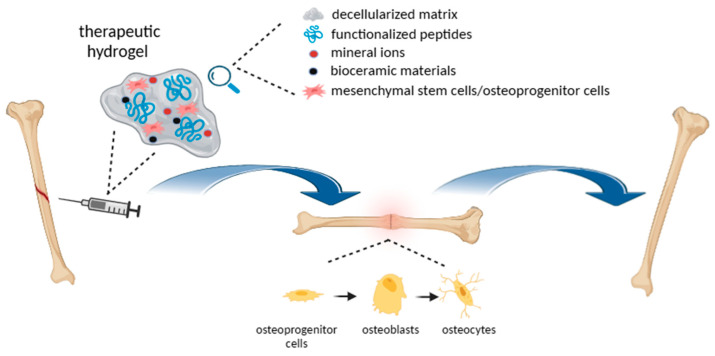
Schematic representation of hydrogel uses in supporting bone regeneration. Image created with BioRender.com.

Figure 2 summarizes the main features involved in the hydrogel-sustained bone repair process, highlighting the key role of an osteoinductive and osteo-permissive environment in sustaining osteoprogenitor cells’ proliferation and differentiation toward osteoblasts and finally osteocytes.

In conclusion, in order to ensure proper bone regeneration, it is crucial to develop scaffolds that could closely mimic the macro- and micro-scale hierarchical architecture of the native tissue. The current trend in biomaterials for bone regeneration research mainly focuses on the definition of composite matrices derived from the combination of inorganic and organic materials, which could assure the obtainment of microscopic features able to improve effective tissue development. In this context, scaffold porosity and roughness represent key requirements. The most recent fabrication approaches rely on the creation of three-dimensional scaffolds with interconnected pores of different dimensions (i.e., 50–150 μm to allow cell colonization and 100–600 μm to facilitate the integration with the host native tissues) whose surface roughness is regulated by a fine-tuning of the starting mixture, as it has been demonstrated that rough surfaces are more effective in supporting bone regeneration compared to smoother ones. Last but not least, fiber alignment also appears to be crucial and thus represents another challenging feature to be reproduced during scaffold production. All these concerns started to be answered by different research groups, allowing a promising step forward for the creation of effective biomaterials to be used to support bone defect resolution [139].

### 4.3. Hydrogel for Articular Cartilage Regeneration

Articular cartilage is a resilient and smooth type of connective tissue whose function is to minimize friction and compressions during movements. Joint cartilage can be easily damaged following physical trauma or in the case of chronic diseases. Since cartilage does not have an intrinsic regenerative potential, the injured tissue cannot naturally recover, thus leading to a worsening in the patient’s quality of life. To date, the available treatments are intended to relieve the symptoms, highlighting the need to develop new therapeutic approaches able to support tissue regeneration. In this context, due to their physical and mechanical properties, hydrogels represent an interesting biomaterial to support damaged cartilage recovery, along with their ability to adapt to the damaged area when injected in situ [127,140,141,142].

Several naturally derived hydrogels proved to support chondrogenesis and cartilage healing [143]. Due to its biocompatibility, bioactivity, and non-immunogenicity, along with its role in maintaining chondrogenic phenotype, hyaluronic acid (HA) has been successfully used to develop hydrogels able to sustain chondrocyte differentiation [144]. Zhang and coworkers achieved hyaline cartilage repair in an in vitro model by synthesizing an injectable collagen type I (Col)-hyaluronic acid (HA) hydrogel loaded with bone marrow mesenchymal stem cells (BMSCs) that successfully differentiated into chondrocytes [145]. Another interesting approach to cartilage regeneration is represented by the development of a hydrogel-based bioink composed of HA and alginate co-printed with polylactic acid (PLA) that has been demonstrated to increase the chondrogenic markers’ gene expression [146]. HA has also been combined with silk fibroin (SF) in composite hydrogels, providing adequate mechanical strength and a slower rate of degradation along with tunable stiffness and viscoelasticity, finally resulting in a scaffold able to promote the deposition of tissue-specific ECM [147].

HA is not the only GAG used to develop chondroinductive hydrogels; as a matter of fact, several authors developed chondroitin sulfate (CS)-based matrices able to support in vitro cartilage regeneration [148,149].

Hydrogels loaded with appropriate cellular populations represent a great challenge for researchers. MSCs, human articular chondrocytes (hACs), and human chondroprogenitor cells (hCPCs) have been investigated as an interesting approach to improve articular cartilage healing. Among these cells, MSCs received great attention due to their proven ability to promote the formation of hyaline-like persistent cartilage [150,151]. Different research groups successfully developed hydrogels embedding different bioactive compounds (i.e., transforming growth factor β3 (TGF-β3), bone morphogenic protein 2 (BMP2), and stromal cell-derived factor 1α (SDF-1α)) intended to sustain MSC differentiation towards a chondrogenic phenotype [152,153], while Scalzone and colleagues designed a thermo-sensitive chitosan hydrogel able to sustain MSCs and chondrocyte co-culture [154].

Hydrogels for cartilage regeneration can also act as anti-inflammatory compound delivery systems, ensuring their controlled release at the damaged site [155,156,157]. As it is known that inflammatory response is critical during the development of degenerative damages, Zhu and coworkers developed a multifunctional thermo-sensitive hydrogel able to remove reactive oxygen and nitrogen species, thus promoting the repolarization of macrophages [158], while Sang’s research group designed a thermosensitive, injectable hydrogel able to release chondrocyte-derived exosomes, achieving a positive regulation of chondrocyte proliferation, migration, and differentiation, as well as an effective macrophage polarization from M1 to M2 [159].

**Figure 3:**
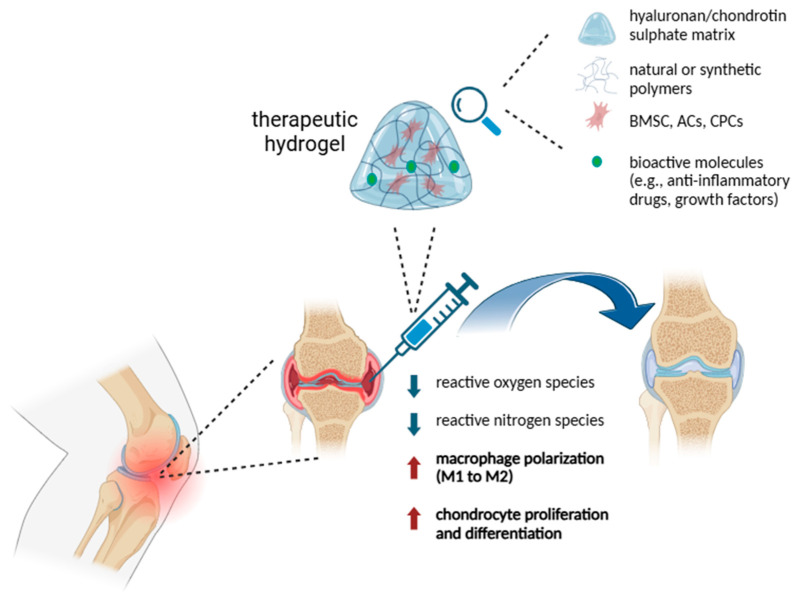
Schematic representation of hydrogel uses in supporting articular cartilage regeneration. BMSC = bone marrow mesenchymal stem cells, ACs = articular chondrocytes, CPCs = chondroprogenitor cells. Image created with BioRender.com.

Figure 3 summarizes the main features involved in the hydrogel-sustained articular cartilage healing process.

Also, with respect to cartilage, it is evident that proper tissue regeneration strongly relies on the development of scaffolds able to closely mimic the original tissue structure both at the macro- and micro-scale level. This challenge has been recently addressed by the development of scaffolds characterized by engraved grid patterns, which have proven to be able to support chondrocyte and mesenchymal cell differentiation, as well as the vertical alignment of the newly produced collagen fibers. Furthermore, since also in cartilage repair scaffold porosity is known to play a crucial role, several researchers started to investigate the combination of pore size and geometry in order to offer the different cell populations recruited the optimal three-dimensional environment to spread and differentiate, as it is known that smaller pores (100–200 μm diameter) promote chondrocyte differentiation, while larger ones (300 μm diameter) are better at supporting mesenchymal cell differentiation toward a chondrogenic phenotype. Again, in the last years, several research groups started to obtain promising results in addressing the tissue-specific challenges imposed by cartilage scaffold fabrication, supporting the development of superior biomaterials for this tissue regeneration [139].

### 4.4. Hydrogel for Skeletal Muscle Regeneration

Skeletal muscle tissue shows an endogenous regenerative ability that is essential to restore its functions following small injuries. Conversely, when tissue damage is extensive, natural healing responses are not effective, and new treatment approaches, such as bioengineered matrices able to support skeletal muscle recovery, are needed.

As previously discussed for their use in supporting other tissues’ regeneration, also in this case different approaches (i.e., stem cells or bioactive molecules delivery, optimization of the scaffold properties) have been developed [160,161,162]. In addition to the traditional approaches, other innovative methods have been successfully tested, such as the use of hydrogel scaffolds to deliver viral or nonviral vectors encoding specific transgenes useful to sustain muscle repair [163,164]. In particular, Doukas and coworkers developed collagen-based matrices able to deliver transgenes intended to support myotube regeneration in a skeletal muscle wound model [164]. Moreover, Falcon and colleagues successfully synthesized a polymeric scaffold used to support the delivery and controlled release of a therapeutic plasmid coding for insulin-like growth factor I (IGF-I), on which skeletal myoblasts attach and proliferate, thus supporting the use of this kind of scaffold for skeletal muscle regenerative purposes [163]. The scaffold’s topography represents another interesting issue to be considered while developing matrices able to sustain skeletal muscle regeneration. For this reason, micropatterned substrates, microfiber hydrogels, and 3D scaffolds with anisotropic porosity have been deeply investigated [165,166,167,168,169]. In particular, it has been observed that such engineered matrices are able to support myoblast alignment while supporting satellite cell growth, myogenic protein expression, and myokine production, finally resulting in an in vivo regeneration [170,171].

In addition to the topography of the scaffold, its mechanical properties are also essential in supporting damaged muscle recovery. As demonstrated by several authors, hydrogel stiffness is essential to support myogenesis by preserving mechanotransduction and optimizing metabolites and waste diffusion, while its elasticity is essential in regulating muscle stem cell responses [169,172,173]. As electrical signals play an essential role in the development and functionality of excitable biological tissues, another key feature of muscle tissue is represented by its electroconductivity. Several research groups have already developed hydrogels with tunable electrical properties able to improve muscle precursor cell spreading and differentiation [174,175,176]. However, despite the promising potential of electroconductive hydrogels, their long-term clinical and therapeutic applications have not been fully investigated.

**Figure 4:**
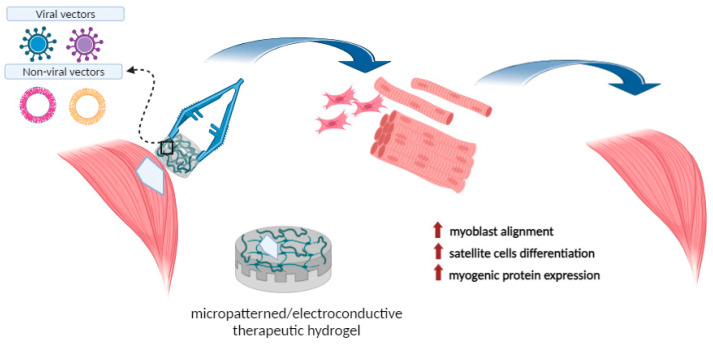
Schematic representation of hydrogel uses in supporting skeletal muscle regeneration. Image created with BioRender.com.

Figure 4 summarizes the therapeutic potential of engineered hydrogels obtained through highly controlled polymerization conditions, allowing a specific micropatterned and electroconductive substrate production in sustaining skeletal muscle regeneration.

In summary, biomaterials intended for the support of skeletal muscle regeneration need to mimic the spatial organization of the native tissue, requiring ordered fiber deposition in order to guide myoblast alignment and maturation. Recent advancements in fabrication techniques have also allowed researchers working in the skeletal muscle regeneration field to address this crucial challenge, thus fostering the development of new scaffolds with a complex hierarchical organization able to support myoblast differentiation towards a striated phenotype [139].

### 4.5. Hydrogels for Cardiac Muscle Regeneration

As well as the skeletal muscle, the cardiac muscle could also undergo pathological damage, needing prompt repair to sustain the heart’s physiological activity. Due to their widely tunable properties, hydrogels represent a very promising solution in the field of cardiac regeneration.

To obtain hydrogels able to sustain cardiac tissue repair, different approaches have been proposed, such as the use of a biocompatible matrix to sustain stem cell delivery at the injury site or hydrogel functionalization with bioactive compounds able to sustain angiogenesis [177,178].

An alternative solution to these injectable matrices is represented by the development of implantable cardiac patches, biocompatible devices that can be composed of different natural substrates, such as decellularized ECM, polysaccharides (e.g., chitosan), and peptides (e.g., collagen, gelatin, fibrin, and silk fibroin), synthetic polymers, or hybrid materials [177,179,180]. Such bioengineered systems have already been successfully used to deliver cells (MSCs or induced pluripotent stem cell-derived cardiomyocytes (hiPSC-CMs)) and therapeutic compounds (i.e., hepatocyte growth factors, insulin-like growth factor-1, VEGF, bFGF) to assist heart tissue regeneration [177,181,182,183,184,185].

As previously discussed for skeletal muscle, electrical activity is also essential in the context of cardiac functionality, and the ability to re-establish the electrical coupling within the damaged tissue is an essential goal in developing biomaterials for cardiac regeneration. Promising results have already been obtained by different authors, leading to the production of electrically conductive hydrogels able to improve cardiac tissue functions and healing [186,187,188]. However, as for hydrogels intended to support skeletal muscle regeneration, preclinical studies are needed to characterize the long-term effects and stability of these novel biomaterials.

**Figure 5:**
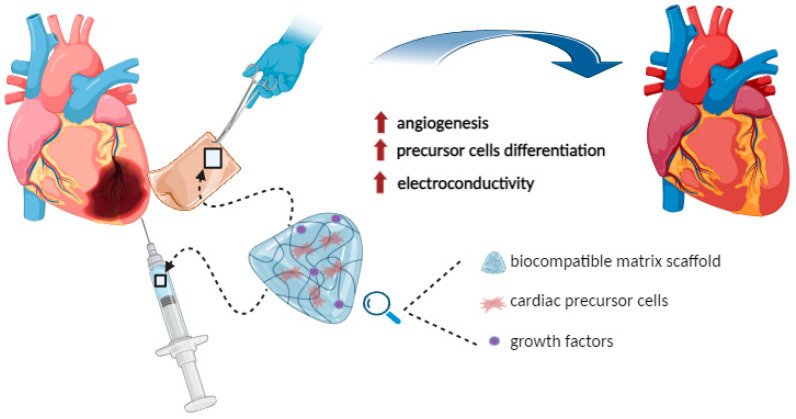
Schematic representation of hydrogel uses in supporting cardiac muscle regeneration. Image created with BioRender.com.

Figure 5 summarizes the principal features of hydrogel use in supporting cardiac regeneration, highlighting the different ways in which such promising bioengineered substrates can be used in clinical contexts (i.e., injectable form or implantable patches).

To sum up, the main challenge in developing successful biomaterials to support heart tissue regeneration is represented by the synthesis of a viscoelastic matrix able to ensure the electrical connectivity needed for the cyclical beating. Also, in this case, the optimal scaffold should closely reproduce the hierarchical complexity of the native tissue, which is essential for cardiac biomechanics. In recent years, several progresses have been made in this field, with the successful development of highly interconnected three-dimensional porous biomaterials with specific square grid patterns, allowing cardiomyocyte differentiation, as well as ensuring their electrical connection in a functional syncytium [139].

## 5. Conclusions

Biomaterial-based approaches have the potential to enhance and solve many drawbacks of the existing approaches for organ and tissue regeneration. In particular, hydrogels, thanks to their wide tunability in terms of physical and mechanical properties, represent the most interesting choice to sustain the restoration of damaged tissue integrity and functionality. Considering the progressive aging of the population and the known effects of such physiological processes on human tissues and organs, tissue engineering represents a valuable alternative to the continuous shortage of suitable donors for grafting. The successful design of bioengineered scaffolds for biomedical applications strongly resides in a deep understanding of the physical and mechanical properties of the biological material to be replaced, as well as in the technical competencies needed to tailor the synthesis of the biological substitute according to the intended use. In this context, hydrogels represent a promising opportunity, as they can be easily produced in the desired size and shape, adapting their physical and mechanical behavior to the surrounding environment. Last but not least, another key advantage of such scaffolds is represented by their biocompatibility and biodegradability, two essential features for any material that should be implanted in a living organism with the aim of supporting cell spreading and proliferation while avoiding exaggerated inflammatory responses and rejection.

Despite the main advantages offered by hydrogel matrices for the development of scaffolds for tissue engineering purposes, many challenges remain to be addressed, especially in terms of tissue-specific constraints. The continuous advancements in the field of biomaterials manufacture (e.g., suitability of starting materials and fabrication techniques) along with the increasing knowledge about native tissues’ biological complexity led to the creation of innovative scaffolds with promising prospects for clinical translation and personalized medicine approaches.

In this context, smart hydrogels, namely hydrogels able to adapt and respond to specific environmental stimuli (e.g., pH, temperature), represent a very interesting solution, which could be further customized, taking advantage of their ability to undergo in situ gelation, to develop substituents showing the most suitable features to support a successful regeneration of damaged tissues.

Even if several promising results have been obtained with both in vitro and in vivo models, the way for the creation of commercial solutions is still long and complex, mainly because of the difficulties in reproducing such complex features over a large area for scaffolds intended for relevant clinical use. Moreover, it should be remembered that, in natural tissues, cells dynamically interact with their surrounding environment, so any newly developed innovative scaffold should be able to spatially and temporally guide cell fate and function in order to restore the original homeostasis.

Finally, to accelerate the translation of the promising experimental results already available into clinical practice, a huge effort in the manufacturing process, ensuring new solutions’ compliance with regulatory requirements, and well-designed preclinical and clinical trials are mandatory.

## Figures and Tables

**Table 1:** Elastic modulus of different tissues.

Biological Tissue	Elastic Modulus
Skin	4.6–20 MPa
Cartilage	10–20 kPa
Bone	1–20 GPa
Heart	30–400 kPa
Skeletal Muscle	20–100 kPa

**Table 2:** Advantages and disadvantages of different types of hydrogels used for tissue engineering applications.

Type	Examples	Advantages	Disadvantages	References
Synthetic	poly(vinyl alcohol) (PVA), poly(ethylene glycol) (PEG), poly(ethylene oxide) (PEO), poly(2-hydroxyethyl methacrylate) (PHEMA), poly(acrylic acid) (PAA), poly(acrylamide) (PAAm), etc.	Excellent durability and reproducibility, high tunability of their mechanical properties	Low bioactivity, possible cytotoxic effects and immune rejection	[68]
Natural	**Protein based**: Collagen, elastin, fibrin, gelatin, silk fibroin **Polysaccharide based**: Glycosaminoglycans, alginate, chitosan **Decellularized hydrogels**: Decellularized ECM from different tissues	Biocompatibility, biodegradability, high bioactivity, low/absent toxicity, high tunability of their characteristics, high retention of growth, and differentiation factors	Poor mechanical stability, batch variability, poor stability over a long period of time, possible lack of reproducibility, limited applicability in terms of 3D printing	[68,69]
Hybrid	Combination of natural and synthetic (e.g., collagen, elastin, fibrin, gelatin, silk fibroin, GAGs, alginate, chitosan, decellularized tissues, poly(lactic acid) (PLA), poly(ε-caprolactone) (PCL), poly(glycolic acid) (PGA) and copolymers, poly(ethylene glycol) (PEG), and poly(vinyl alcohol) (PVA))	Remarkable thermodynamic stability, elevated capacity of solubilization, tunable mechanical properties, heterogeneous structure allowing cell adhesion and spreading, responsiveness, biocompatibility, biodegradability, and non-immunologic response	The incorrect combination of materials could result in incompatible and non-functional hydrogels	[70,71,72]

**Table 3:** Brief description of the distinctive features of the currently available hydrogel fabrication methods.

Fabrication Methods	Key Features	Advantages	Disadvantages	Examples
Emulsification	It produces minute hydrogel droplets by mixing multi-phased solutions with a hydrophobic phase. Hydrogel droplet size could be easily tailored by modifying the precursor’s viscosity and mixing intensity. Agglomeration could be prevented by adding surfactants to limit surface tension.	Possibility to obtain cell-laden scaffolds by simply adding cells to the mixing phase.	Only spherical particles could be produced, with a wide particle size distribution.	Natural (chitosan, collagen, agarose, alginate) and synthetic (polylactic acid, polylactic-co-glycolic acid) polymers can be used to encapsulate cells to develop a controllable environment for differentiation.
Freeze-drying	Polymer and solvent are added to a water solution undergoing rapid cooling in a temperature range, causing thermal instability within the structure. This freeze-dried construct is then subjected to partial vacuum, allowing solvent to evaporate while building porous scaffolds.	Possibility to create porous matrices. Possibility to use water and ice crystals instead of organic solvents during the fabrication process.	Difficulty in tuning pores size and long processing time, resulting in relatively poor mechanical features due to matrix collapse following the scaffold–air interface tension changes during solvent evaporation.	Natural (collagen, chitosan, agarose, silk proteins), synthetic (poly(ethylene glycol), poly(L-lactic acid)) and composite (polylactic-co-glycolic acid-poly(propylene fumarate), collagen-chitosan) polymers can be used. Collagen–chitosan scaffolds crosslinked with glutaraldehyde were tested for adipose tissue engineering, while agarose scaffolds with linear porous channels showed promising results in supporting azonal regeneration.
Porogen leaching	Salt particles are mixed with a solvent and transferred to a mold of the desired shape, which undergoes freeze-drying, allowing solvent evaporation, and subsequent leaching of the trapped salt particles within the network.	Fair reproducibility and lack of sophisticated fabrication apparatus. Possibility to control pore size and quantity by adjusting the nature and quantity of porogen used. Possibility to develop scaffolds with dual porosity.	Need for long soaking in water to remove salts and solvents used during the fabrication. Potential presence of residual porogen and/or solvent within the network, forming defective pore structures.	Natural (collagen) and synthetic (polylactic-co-glycolic acid, poly(lactic acid)) can be used to obtain composite scaffolds.
Gas foaming	Effervescent salt particles are mixed into a polymer gel cast in a mold where gas bubbles are generated with either chemical or physical methods, decreasing the polymer solubility. Consequently, the pressure causes gas nucleation and the formation of interconnecting pores, finally resulting in highly porous foam scaffolds without the use of organic solvents, which can have toxic effects or induce inflammatory responses.	Possibility to obtain scaffolds with high porosity (up to 90–93%). Possibility to obtain macroporous structures with a homogeneous pore size ranging between 100 and 200 μm. Possibility to maintain the bioactivity of the molecules embedded into the matrix.	Formation of skimming film layers on the scaffold surface, which needs to be removed. Poor interconnectivity of the porosity.	Mixtures of poly(lactic acid) and ammonium bicarbonate, polylactic-co-glycolic acid and citric acid, poly(acrylic acid/acrylamide) and sodium bicarbonate or poly(ethylene glycol) and sodium bicarbonate have been successfully used to produce highly porous foams.
Electrospinning	A high voltage current is applied to the syringe pump filled with the polymeric material, which jets the solution out of the nozzle tip as thin filaments collected by a rotating collector.	Possibility to incorporate proteins and/or growth factors in poorly biocompatible synthetic polymers to improve their whole features. Possibility to obtain submicrometric porous fibrous hydrogels, assuring improved cell–scaffold interactions.	Difficulties in generating scaffolds with complex structures and homogeneous pore distribution. Inability to generate three-dimensional scaffolds.	Natural (collagen, chitosan, silk fibroin, chitin) and synthetic (poly(lactic acid), poly(glycolic acid), poly(lactic-co-glycolitic acid), polycaprolactone, poly(ethylene oxide), polyvinyl alcohol) polymers can be used to develop scaffolds for tissue engineering applications.
Three-dimensional printing	It represents an innovative strategy to fabricate matrices with precise size and shape, in which it is possible to precisely position biologics, such as living cells, and ECM components according to a hierarchical organization.	Possibility to replace the classical two-dimensional (2D) models in which cells are grown as monolayers without reproducing the dynamic and complex cell–cell and cell–matrix interactions, thus improving in vivo physiological interactions in artificial multicellular tissues/organs.	Difficulties in the identification of the printable materials that should possess structural stability in biological environments, a degradation rate congruent with tissue regeneration, and non-toxic features.	Natural (alginate, collagen, silk, dextran, gelatin, fibrin, agarose, gellan gum, hyaluronic acid, decellularized matrices), synthetic (pluronics, poly(ethylene glycol), polycaprolactone) and composite (agarose/chitosan, alginate/gelatin, fibrinogen/gelatin, hydroxyapatite/gelatin) polymers can be used to obtain cell-laden scaffolds with predefined external shape and internal morphology.
Photolithography	This is a two-step technique in which a masked photosensitive polymer modelled in the desired shapes and sizes is exposed to UV radiation to allow the photopolymerization, while the unreacted substrate is eliminated through solvent washing.	Possibility to synthesize polymeric 3D scaffolds with tunable alignment patterns. Possibility to create scaffolds in which cells are encapsulated within photocrosslinkable polymers. Possibility to conjugate chemical moieties to the hydrogel matrix in spatially controlled manner.	Need of photo-crosslinkable polymers and photoinitiators that could be cytotoxic. Possible noxious effects of UV radiation on cell behavior. Need for further assembly of the obtained two-dimensional structures in order to obtain three-dimensional scaffolds.	Acrylic monomers, acrylate-functionalized polymers (poly(ethylene glycol) diacrylate, acrylated gelatin, acrylated alginate) or vinyl-functionalized macromolecules have been successfully photopolymerized to obtain scaffold with arbitrary geometries.
Sol–gel technique	Scaffolds are prepared by dissolving organic or inorganic metal compounds in a solvent. The resulting solution is subjected to cycles of hydrolysis and polymerization reactions, allowing the formation of a colloidal suspension (sol) that is cast into a mold to achieve a gel structure that is converted into a dense ceramic or glass material through further drying and heat treatments.	Possibility to obtain scaffolds with high chemical homogeneity by working with low processing temperatures. Possibility of controlling particle size and morphology.	High cost of raw materials. Large shrinkage during processing. Possible health hazards deriving from the long processing time involving organic solutions.	Natural (alginate, gelatin, cellulose) and synthetic (poly(ethylene glycol), polyvinyl alcohol) polymers can be used to obtain porous scaffolds.

**Table 4:** Brief description of the most important cellular mechanosensors and their mechanism of action.

Membrane-Located Mechanosensors	Mode of Action
Cadherins	They act as tension transducers, transmitting mechanical stimuli to the cytoskeletal actin filaments.
Desmosomal cadherins	They assure mechanical integrity to the junctional complex by acting on cytoskeletal intermediate filaments.
Connexins	They enhance cell–cell communications at gap junction level, assuring a syncytium-like behavior.
Integrins	They act as tension transducers, transmitting mechanical stimuli to the cytoskeletal intermediate filaments.
Focal adhesions	They strengthen the actomyosin cytoskeletal network thanks to mechanical stimulation-induced conformational changes.
Transient receptor potential vanilloid 4 (TRPV4) channels	They allow calcium influx and signaling in response to a direct mechanical stimulation.
Piezo1 channels	They allow calcium movement, influencing cytoskeletal rearrangement.
Very large G protein-coupled receptor 1 (VLGR1)	They regulate focal adhesion assembly and disassembly and stimulate cell migration.
**Intracellular Mechanosensors**	**Mode of Action**
Linker of the nucleoskeleton and cytoskeleton (LINC) complex	It transmits mechanical stimuli between the nucleoskeleton and the cytoskeleton.
Yes-associated protein and transcriptional coactivator with PDZ-binding motif (YAP/TAZ)	They alter their localization between the cytoplasm (inactive state) and the nucleus (active state) in response to mechanical stimuli, thus regulating cell growth, differentiation, and migration.
Rho-associated kinase (ROCK)	It stabilizes the actin cytoskeleton, favors actin/myosin crosslinking, and enhances actomyosin contractility.
mDia	It promotes actin cytoskeleton assembly by favoring its nucleation and polymerization.
Focal adhesion kinase (FAK)	It transduces mechanical stimuli to the myosin cytoskeleton, finally regulating cell differentiation by acting upstream on the Rho/ROCK pathway.
Mixed lineage kinases (MLKs)	Under compression stimulation, they activated the downstream mitogen-activated protein kinase (MAPK) pathway, a key regulator of cell differentiation.
Wnt/β-catenin pathway	Mechanical stimuli, like shear stress or tension, lead to integrin activation and β-catenin accumulation and/or Wnt ligand release, finally resulting in the regulation of cell differentiation and homeostasis.
